# The Natural History of Spinocerebellar Ataxia Type 3 in Mainland China: A 2-Year Cohort Study

**DOI:** 10.3389/fnagi.2022.917126

**Published:** 2022-07-05

**Authors:** Yun Peng, Linliu Peng, Zhao Chen, Huirong Peng, Puzhi Wang, Youming Zhang, Yangping Li, Chunrong Wang, Yuting Shi, Xuan Hou, Zhe Long, Hongyu Yuan, Na Wan, Linlin Wan, Keqin Xu, Lijing Lei, Shang Wang, Lang He, Yue Xie, Yiqing Gong, Qi Deng, Guangdong Zou, Zhichao Tang, Lu Shen, Kun Xia, Rong Qiu, Thomas Klockgether, Beisha Tang, Hong Jiang

**Affiliations:** ^1^Department of Neurology, Xiangya Hospital, Central South University, Changsha, China; ^2^Department of Radiology, Xiangya Hospital, Central South University, Changsha, China; ^3^Department of Human Genetics, Emory University School of Medicine, Atlanta, GA, United States; ^4^Department of Pathology, Xiangya Hospital, Central South University, Changsha, China; ^5^Department of Neurology, The Second Xiangya Hospital, Central South University, Changsha, China; ^6^Laboratory of Medical Genetics, Central South University, Changsha, Hunan, China; ^7^National Clinical Research Center for Geriatric Diseases, Xiangya Hospital, Central South University, Changsha, China; ^8^Key Laboratory of Hunan Province in Neurodegenerative Disorders, Central South University, Changsha, China; ^9^Hunan International Scientific and Technological Cooperation Base of Neurodegenerative and Neurogenetic Diseases, Changsha, China; ^10^School of Computer Science and Engineering, Central South University, Changsha, China; ^11^Department of Neurology, University of Bonn, Bonn, Germany; ^12^German Center for Neurodegenerative Diseases (DZNE), Bonn, Germany

**Keywords:** spinocerebellar ataxia type 3, *ATXN3*, natural history, disease progression, Machado-Joseph disease (MJD)

## Abstract

**Objective:**

The natural history of spinocerebellar ataxia type 3 (SCA3) has been reported in several populations and shows heterogeneity in progression rate and affecting factors. However, it remains unexplored in the population of Mainland China. This study aimed to identify the disease progression rate and its potential affecting factors in patients with SCA3 in Mainland China.

**Participants and Methods:**

We enrolled patients with genetically confirmed SCA3 in Mainland China. Patients were seen at three visits, i.e., baseline, 1 year, and 2 years. The primary outcome was the Scale for the Assessment and Rating of Ataxia (SARA), and the secondary outcomes were the Inventory of Non-Ataxia Signs (INAS) as well as the SCA Functional Index (SCAFI).

**Results:**

Between 1 October 2015, and 30 September 2016, we enrolled 263 patients with SCA3. We analyzed 247 patients with at least one follow-up visit. The annual progression rate of SARA was 1.49 points per year (SE 0.08, 95% confidence interval [CI] 1.33–1.65, *p* < 0.0001). The annual progression rates of INAS and SCAFI were 0.56 points per year (SE 0.05, 95% CI 0.47–0.66, *p* < 0.001) and −0.30 points per year (SE 0.01, 95% CI −0.33∼-0.28, *p* < 0.001), respectively. Faster progression in SARA was associated with longer length of the expanded allele of *ATXN3* (*p* < 0.0001); faster progression in INAS was associated with lower INAS at baseline (*p* < 0.0001); faster decline in SCAFI was associated with shorter length of the normal allele of *ATXN3* (*p* = 0.036) and higher SCAFI at baseline (*p* < 0.0001).

**Conclusion:**

Our results provide quantitative data on the disease progression of patients with SCA3 in Mainland China and its corresponding affecting factors, which could facilitate the sample size calculation and patient stratification in future clinical trials.

**Trial Registration:**

This study was registered with Chictr.org on 15 September 2015, number ChiCTR-OOC-15007124.

## Introduction

Spinocerebellar ataxia type 3 (SCA3), also known as Machado-Joseph disease (MJD), is the most common autosomal dominant inherited ataxia worldwide (Chen et al., 2018; Klockgether et al., 2019). It is caused by the expansion of a CAG repeat in the *ATXN3* gene, leading to a slowly progressive cerebellar ataxia with other various symptoms (Costa Mdo and Paulson, 2012). As promising treatments for SCA3 are being developed, it is vital to address the disease progression of SCA3 and identify the potential factors affecting disease progression. During the past two decades, several natural history studies of SCA3 have been conducted in Europe (Klockgether et al., 1998; Schmitz-Hubsch et al., 2008a; Jacobi et al., 2011, 2015, 2018; Tezenas Du Montcel et al., 2012; Diallo et al., 2018), America (Franca et al., 2009; Ashizawa et al., 2013; Kuo et al., 2017), and Asia (Lee et al., 2011; Lin et al., 2019). These studies have shown variance in results of disease progression rate and affecting factors in different areas and populations (Diallo et al., 2021). Till present, there is no report about the natural history of SCA3 in Mainland China. In this study, we longitudinally investigated a cohort of patients with SCA3 in Mainland China. We aimed to explore the disease progression of SCA3 and identify factors that affect disease progression.

## Participants and Methods

### Study Design and Participants

We performed a 2-year longitudinal cohort study of patients with SCA3 in Xiangya Hospital, Central South University, China. Patients were eligible when all the following inclusion criteria were met: (1) with pathogenic allele (CAG repeats ≥ 55) in *ATXN3* (Lieberman et al., 2019); (2) with the Scale of Assessment and Rating of Ataxia (SARA) ≥3, defined as the ataxic stage (Maas et al., 2015); and (3) be able to understand and provide written informed consent to participate in the study. Patients with neurological symptoms and signs which could not be attributed to SCA3 or with other neurological and psychiatric disorders were excluded in this study.

Patients were consecutively recruited within a predetermined period between 1 October 2015 and 30 September 2016. Patients were followed annually for 2 years, and visits were done in a time window of ± 1 month around the scheduled time. All the follow-ups of the patients were accomplished by October 2018. Every patient was seen by the same investigator at all visits. All investigators were well trained in using the clinical scales and passed the unified qualification test prior to participating in this study to reduce bias.

We estimated a sample size of 150 patients with SCA3 based on the previous literature of natural history studies in SCA3 (Jacobi et al., 2011, 2015). However, we included all available participants with SCA3 between 1 October 2015 and 30 September 2016, in Xiangya Hospital, Central South University, China.

This study was approved by the Ethics Committee of Xiangya Hospital, Central South University in China (number 201412402). This study was registered with Chictr.org (number ChiCTR-OOC-15007124). All patients provided written informed consent to participate in the study.

### Outcome Measures

The detailed demographic data were obtained at baseline, and the clinical examination and evaluations were performed at every visit. For assessing the disease progression, we used the SARA as the main outcome measure. Meanwhile, we used the Inventory of Non-Ataxia Signs (INAS) and the SCA Functional Index (SCAFI) as the secondary outcome measures. SARA is a validated scale for evaluating ataxia symptoms (Schmitz-Hubsch et al., 2006); SARA scores range from 0 to 40, with 0 representing no ataxia and 40 representing the most severe ataxia. Prior to this study, we held a session with Dr. Thomas Klockgether and Dr. Alexandra Durr to harmonize the SARA scoring in Changsha to reduce the bias of assessment. INAS is a validated instrument for evaluating non-ataxia signs (Jacobi et al., 2013); INAS count ranges from 0 to 16, with 0 representing no non-ataxia symptoms, and 16 representing all items of non-ataxia symptoms included in INAS. The SCAFI is a validated composed index of movement speed (Schmitz-Hubsch et al., 2008b,^2010^), which includes a timed 8-m walk (8MW), the 9-hole peg test (9HPT), and the rate of “PATA” repetition over 10 s (PATA). Specifically, all SCAFI components (8MW and 9HPT after inversion to their reciprocals) were first transformed into Z-scores expressed as standard deviation (SD) from baseline mean, and then the SCAFI was calculated as the mean of its component Z-scores. A higher SCAFI score means a better movement function.

### Genetic Analysis

We extracted DNA from ethylenediaminetetraacetic acid blood samples and tested the length of CAG repeats of *ATXN3* in the Center for Medical Genetics, Central South University, using previously established methods (Peng et al., 2014).

### Statistical Analyses

The data for quantitative variables and categorical variables were described as mean (standard deviation) and frequency, respectively. The standardized response mean (SRM) was calculated as the mean annual change in the corresponding score of scale divided by the SD of the annual change. We analyzed the yearly progression for SARA, INAS, and SCAFI with the linear mixed model (restricted-maximum-likelihood method, with random effects on intercept and slope). The time variable was the year since inclusion. The family was considered a random effect. The unstructured covariance was used as the repeated covariance type. First, we tested a linear and quadratic effect of time and chose the linear model according to the Akaike information criterion (AIC). Second, we tested the effects of demographic and disease-related factors on progression rates. Specifically, we used the SARA score, INAS count, or SCAFI as dependent variables and used the repeat length of expanded allele, repeat length of normal allele, gender, age at baseline, age at onset, and disease duration at baseline as independent variables. The influence of these variables on the progression rate of SARA, INAS, or SCAFI was tested *via* interactions between corresponding variables and time variables. In addition, we included the baseline scores as main effects. Independent factors that were significant in the univariate analysis were included in a multivariate model. In this step, numerical variables were mean centered to help interpretation, and the model was also adjusted on family effect. Third, based on the progression rate of SARA identified in this study, we calculated the sample size for a two-armed clinical trial of treatments (Green and Macleod, 2016), which could detect a reduction in progression as assessed with SARA with different efficacies for the observation periods of 1 year and 2 years.

All statistical analyses were performed using IBM SPSS Statistics version 25 (Armonk, NY, United States) except for the sample size calculation, which was performed using the R software (version 4.0.4; Development Core Tean, 2021), “lme4” package (Bates et al., 2015), and “simr” package (Green and Macleod, 2016). All tests were two-sided, and the level of significance was set at 0.05.

## Results

**FIGURE 1:**
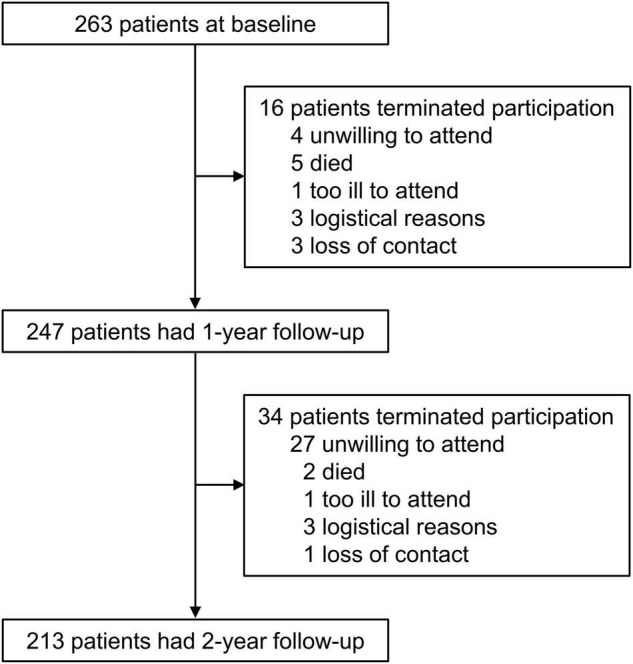
Study profile. Data show the patient number at each visit and the reasons for the dropout.

Between 1 October 2015 and 30 September 2016, we enrolled 263 patients with SCA3. These patients were from 21 provinces, centrally administered municipalities, or autonomous regions, and the majority (80.6%) of them were from Hunan Province in southern China (Supplementary Figure 1). We analyzed the disease progression in a subgroup of 247 patients with at least one follow-up visit. Figure 1 shows detailed information about the number of patients at each year’s visit and the reasons for the dropout of patients. Table 1 shows the demographic and clinical data, with no difference between the whole cohort and the subgroup for analysis. Detailed information about the scores at each visit is shown in Supplementary Table 1, and further information about the subitem of SARA and INAS is shown in Supplementary Tables 2, 3.

**TABLE 1:** Baseline characteristics of the SCA3 cohort.

	Full cohort	Subgroup with at least one follow-up	Statistics (χ^2^ or *U*)	*P*
No.	263	247	NA	NA
No. of families	165	155	NA	NA
Women (%)	138 (52.5%)	131 (53.0%)	0.02	0.898
Age at baseline (years)	44.67 ± 11.29	44.42 ± 10.81	32,140.00	0.838
Age at onset (years)	36.29 ± 10.22	36.15 ± 9.86	32,298.50	0.913
Disease duration (years)	8.38 ± 5.08	8.27 ± 5.10	31,985.50	0.765
Length of expanded allele (repeat units)	71.88 ± 3.43	71.83 ± 3.31	32,452.50	0.986
Length of normal allele (repeat units)^a^	19.98 ± 5.95	20.00 ± 5.99	32,224.50	0.999
SARA score	15.61 ± 7.94	15.45 ± 7.83	32,049.50	0.795
INAS count	4.84 ± 2.35	4.86 ± 2.36	32,347.50	0.936
SCAFI score^b^	−0.35 ± 1.20	−0.32 ± 1.19	19,581.00	0.857

*Quantitative variables are given as mean ± SD; categorical variables as given as numbers (frequency); SARA, scale for the Assessment and Rating of Ataxia; INAS, inventory of non-ataxia signs. Length of expanded allele or normal allele refers to the CAG repeats in the ATXN3 gene.*

*^a^There was a patient with homozygous ATXN3 mutation (with 67 CAG repeats on both allele), and this patient was excluded when calculating the mean and SD for length of normal allele (repeat units).*

*^b^SCAFI score was available in 204 patients of the full cohort, and 194 patients of the subgroup with at least one follow-up. The Mann-Whitney U test was used for the comparation of quantitative variables between groups, including age at baseline, age at onset, disease duration, length of expanded allele, length of normal allele, SARA score, INAS count, and SCAFI. The χ^2^ test for the comparation of gender between groups. After Bonferroni correction, only P-values < 0.0056 were considered significant.*

**FIGURE 2:**
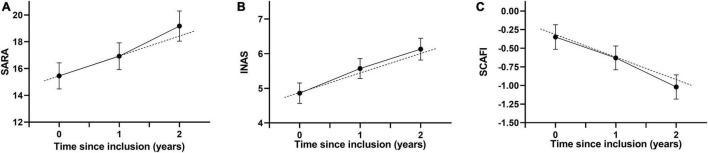
Progression of SARA **(A)**, INAS **(B)** and SCAFI **(C)** in patients with SCA3. Data are mean (95% CI). Dots indicate the mean values. The continuous lines show the changes of recorded values, and the dashed lines shows the estimated progression based on the linear mixed model. SARA, scale for the assessment and rating of ataxia; INAS, inventory of non-ataxia signs; SCAFI, SCA Functional Index.

**FIGURE 3:**
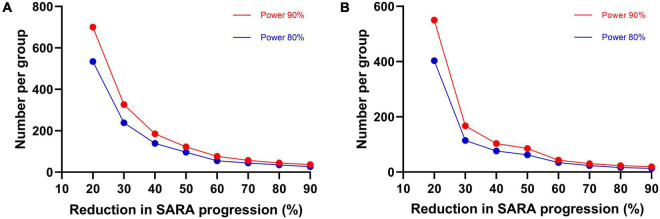
Sample size estimates. Required sample size per group in two-armed clinical trial for 1-year **(A)** and 2-years duration **(B)**, to detect various differences in SARA progression as a function of treatment efficacy and a statistical power of 80 and 90%.

We found that the progression of SARA, INAS, and SCAFI was best fitted with a linear model. The annual progression of SARA is 1.49 points per year (SE 0.08, 95% confidence interval [CI] 1.33–1.65, *p* < 0.0001), the progression of INAS is 0.56 points per year (SE 0.05, 95% CI 0.47–0.66, *p* < 0.001), and the progression of SCAFI is −0.30 points per year (SE 0.01, 95% CI −0.33∼-0.28, *p* < 0.001; Figure 2). Based on the progression rate of the SARA score, we further calculated the sample size needed for two-armed clinical trials of 1 year and 2 years with different effect sizes at 80 and 90% power (Figure 3). For a potential treatment efficacy of 50% reduction in SARA progression rate at the 80% power, 192 (96 per group) patients would be needed in a 1-year clinical trial and 124 (62 per group) patients would be needed for 2-year clinical trial.

To identify the independent factors that were associated with the progression of SARA, INAS, and SCAFI, we first tested the candidate factors in the univariate analysis and further in the multivariate analysis. In the univariate analysis, factors that were associated with faster progression of SARA score were longer length of expanded allele (0.107 [SE 0.024] per additional repeat unit, *p* < 0.0001), younger age at baseline (−0.027 [SE 0.007] per year, *p* < 0.001), and younger age at onset (−0.030 [SE 0.008] per year, *p* < 0.001; Supplementary Table 4). Further multivariate analysis indicated that the length of the expanded allele was the only independent factor associated with SARA progression, whereas the age at baseline and the age at onset lost significance (Table 2 and Supplementary Table 4). In the univariate analysis for INAS, factors that were associated with faster progression of INAS count were longer length of expanded allele (0.027 [SE 0.014] per additional repeat unit, *p* = 0.049), younger age at onset (−0.010 [SE 0.005] per year, *p* = 0.036), longer disease duration at baseline (0.019 [SE 0.009] per year, *p* = 0.030), and lower INAS at baseline (−0.099 [SE 0.020] per year, *p* < 0.0001; Supplementary Table 5). In the further multivariate analysis, the lower INAS at baseline was the only significant independent factor associated with faster INAS progression (Table 2 and Supplementary Table 5).

**TABLE 2:** Linear mixed-effect modeling for multivariable affecting SARA, INAS, and SCAFI progression.

Response variable	Variables in fixed effect	Estimate	SE	95% CI	*t*	*P*
SARA	Time	1.491	0.079	1.336∼1.646	18.938	4.398e-50
	SARA at baseline	0.999	0.004	0.991∼1.007	245.779	0.000
	Time*Length of expanded allele	0.107	0.024	0.060∼0.154	4.493	1.1e-5
INAS	Time	0.547	0.046	0.457∼0.638	11.898	4.951e-26
	INAS at baseline	0.953	0.023	0.909∼0.998	42.149	1.298e-122
	Time*INAS at baseline	−0.099	0.020	−0.138∼–0.060	−4.999	1e-6
SCAFI	Time	−0.272	0.011	−0.293∼–0.251	−25.391	4.061e-60
	SCAFI at baseline	0.996	0.007	0.982∼1.010	139.931	3.340e-195
	Time*Length of normal allele	0.004	0.002	0.0002∼0.0071	2.060	0.041
	Time*SCAFI at baseline	−0.094	0.009	−0.111∼–0.076	−10.669	9.163e-21

*Estimates derived from the model are given as mean, standard error (SE), and 95% confidence interval (CI); SARA, scale for the Assessment and Rating of Ataxia; SCAFI, SCA Functional Index; INAS, inventory of non-ataxia signs; SE, standard error. Length of expanded allele or normal allele refers to the CAG repeats in the ATXN3 gene. These three linear mixed models were built for exploring the independent factors which affects the SARA, INAS, or SCAFI progression. First, the univariate analysis was done separately for the seven interesting variables: repeat length of expanded allele, repeat length of normal allele, gender, age at baseline, age at onset, disease duration at baseline, and baseline scores of SARA or INAS or SCAFI. Independent factors that were significant in the univariate analysis were included and further tested in a multivariate model. These models also included family as a random effect. The detailed steps and data in constructing these three final models were shown in Supplementary Tables 2–4.*

In the univariate analysis for SCAFI, factors that were associated with a faster decline of SCAFI score were shorter length of normal allele (0.004 [SE 0.002] per additional repeat unit, *p* = 0.036) and higher SCAFI at baseline (−0.094 [SE 0.009] per year, *p* < 0.0001; Supplementary Table 6), both of which remained significant in the further multivariate analysis (Table 2 and Supplementary Table 6).

## Discussion

This study first demonstrated the progression rate of disease severity in a longitudinal cohort of patients with SCA3 in Mainland China. Specifically, a linear progression was observed in ataxia symptoms (assessed by SARA), non-ataxia signs (assessed by INAS), and functional capacity (assessed by SCAFI) in an observation period of 2 years. Based on the progression rate of SARA, we established an estimate of sample size for future clinical trials. In addition, we identified the longer length of expanded allele as an independent factor with faster SARA progression. These findings provide population-specific data for the design of SCA3 clinical trials in Mainland China.

Our findings of the annual SARA progression rate were largely consistent with the long-term findings in the 122 SCA3 patients of the EUROSCA cohort in Europe (1.56, 95% CI 1.42–1.70; Jacobi et al., 2015), the 3-year findings in the 58 patients of France (1.70, 95% CI 1.31–2.09; Tezenas Du Montcel et al., 2012), and the 5-year findings in the 118 patients of Taiwan (1.60, 95% CI 1.33–1.87; Lin et al., 2019), but faster than the 2-year findings in the 138 patients of CRC-SCA in the United States (0.65, 95% CI 0.18–1.12; Ashizawa et al., 2013). Therefore, our findings confirmed that the disease progression rate in patients with SCA3 in Mainland China is similar to patients in Taiwan and European countries. The discrepancy between the results of most studies (including this study) and CRC-SCA might be caused by a more complex population background in the United States and a relatively higher ratio of dropping out in the CRC-SCA study. In addition, our result was also close to recent meta-analysis research in which the pooled rate of annual increase of SARA was 1.41 (95% CI 0.97–1.84; Diallo et al., 2021), and the sample size estimation based on SARA progression in our study was also close to the results of EUROSCA and the meta-analysis (Jacobi et al., 2015; Diallo et al., 2021). Our study further extends the understanding of SCA3 natural history in the major population of the world and suggests that a common clinical trial plan might be feasible in the future international clinical trials of SAC3 in these areas.

Till present, it remains controversial about the factors associated with the progression rate of ataxia in SCA3. In this study, we identified that the longer length of the expanded allele was independently associated with faster SARA progression. Our result is consistent with the recent study in Taiwan (Lin et al., 2019) but not with other previous studies. In the interim 2-year report of EUROSCA, the SARA progression was influenced by the disease duration at inclusion (Jacobi et al., 2011), whereas no factors affecting disease progression were found in the 8-year long-term report of EUROSCA (Jacobi et al., 2015). In addition, there was no factor associated with SARA progression found in a study in France (Tezenas Du Montcel et al., 2012). Interestingly, controversial results were also reported in other SCA3 studies using the International Cooperative Ataxia Rating Scale (ICARS), another validated ataxia rating scale similar to SARA. In a SCA3 cohort in Brazil, no factors were associated with ICARS progression (Franca et al., 2009). In contrast, a recent SCA3 study in the Netherland found that the length of the expanded allele was the major determinants in ICARS progression (Leotti et al., 2021). The causes of the discrepancies among these studies still remain unclear. A possible explanation is the difference in genetic background. The accelerator effect of the expanded CAG repeat of *ATXN3* on ataxia progression may only exist in certain populations. As there is a common genetic background of the population in Mainland China and Taiwan, we could find the expanded CAG repeat as a common factor affecting the ataxia progression rate in SCA3. This hypothesis could be supported by two recent studies, which show the effect of genetic background on disease onset and disease severity in SCA3 (de Mattos et al., 2019; Gan et al., 2020). However, due to high heterogeneity of phenotype in SCA3, more detailed individual–patient data from studies of different ethnicity and population are still needed to verify our hypothesis in the future. The finding of the accelerator effect of the expanded CAG repeat of *ATXN3* on ataxia progression could facilitate the patient stratification in future SCA3 trials in Mainland China.

Multiple non-ataxia signs accompanying ataxia are an important characteristic of SCA3. However, the assessment of non-ataxia signs was rarely adopted in the previous SCA3 natural history studies. In the interim 2-year report of EUROSCA, a linear increase of INAS was reported (0.30 [0.08] per year, mean [SE]) and was faster in women than men (Jacobi et al., 2011). In addition, in the final long-term report of EUROSCA, the increase of INAS reached a plateau toward the end of observation, but the overall increase of INAS was still faster in women than men (Jacobi et al., 2015). In the present 2-year study, we observed a linear progression of INAS, which is slightly faster than the interim report of EUROSCA. However, we did not identify the effect of gender on the progression of INAS, and the only independent factor associated with faster INAS progression is a lower baseline INAS count. As the faster INAS progression is associated with a lower baseline INAS count in our study, it is possible that the increase of INAS would also reach a plateau if the observation continued for a longer time. Therefore, we thought that the different lengths of observation time may cause discrepancy in the increased rate of INAS in these two studies. In addition, we further compared the demographic and clinical characteristics between the male and female participants at baseline (Supplementary Table 7). Interestingly, INAS total score tends to be higher in women than men, although without statistical significance (Supplementary Table 7). It is possible that the potential difference in baseline INAS between men and women might weaken the gender effect on INAS progression in our study. However, more studies are needed to address this issue more clearly in the future.

The SCAFI is designed for testing the functional capacity of walking, hand movement, and speaking (Schmitz-Hubsch et al., 2008b). In a previous study comparing different rating instruments in SCA, SCAFI shares a similarly good validity, reproducibility, and responsiveness as SARA (Schmitz-Hubsch et al., 2010). Consistently, SCAFI is highly associated with the SARA score in this study (Supplementary Figure 2 and Supplementary Table 8). The annual progression rate of SCAFI found in our study is slightly higher than the rate of abbreviated SCAFI (SCAFI-AB) reported in the CRC-SCA study (Ashizawa et al., 2013). This is consistent with that the annual progression rate of SARA in our study is higher than the CRC-SCA study (Ashizawa et al., 2013). However, we also noted the floor effect (Supplementary Figure 2) and a relatively high ratio of missing value of SCAFI (Supplementary Table 1) in our study. Compared with participants with fully investigated SCAFI data, the participants with missing data had a higher baseline SARA score (Supplementary Table 9). Both the floor effect and missing data might cause a bias in the interpretation of SCAFI data in our study. In the 2-year CRC-SCA study, the authors observed a large data variability of a component of SCAFI (PATA test) and mentioned another concern that the devices used to assist walking may complicate the measurement of the 8MW component of SCAFI (Ashizawa et al., 2013). Given that the 2-year follow-up of the CRC-SCA study and our study were relatively short, more long-term studies are needed to determine the application value of SCAFI in SCA3.

The main strengths of this study included the large sample size of SCA3 patients in a single natural history study, which would enable a more robust estimation of progression rate. Our study had some limitations. First, the observation time of 2 years is relatively short for such a long-term disease, which may cause some bias like the interim report of EUROSCA. However, a longer time of observation is very likely to cause a high ratio of dropping out, which may also cause another form of bias. Recently, SARA*^home^*, a newly developed video-based tool for the assessment of ataxia at home, is highly correlated with conventional SARA and very easy to accomplish at home, which might be very helpful in long-term observation and reducing the ratio of dropping out (Grobe-Einsler et al., 2021). Second, we did not collect other longitudinal data like neuroimaging data, and longitudinal biofluid samples, which prevented us from describing a more comprehensive profile of disease progression.

## Conclusion

Our results provide quantitative data on the progression of SCA3 in Mainland China. In addition, our study identified the expanded CAG as a factor for the faster progression of ataxia symptoms. These results could provide useful information for sample size calculation and patient stratification in future SCA3 clinical trials.

## Data Availability Statement

The original contributions presented in this study are included in the article/Supplementary Material, further inquiries can be directed to the corresponding author.

## Ethics Statement

The studies involving human participants were reviewed and approved by the Ethics Committee of Xiangya Hospital, Central South University in China. The patients/participants provided their written informed consent to participate in this study.

## Author Contributions

YP, LP, ZC, BT, and HJ contributed to the conception and design of the study. YP, LP, ZC, HP, PW, YZ, YL, CW, YS, XH, ZL, HY, NW, LW, KX, LL, SW, LH, YX, YG, QD, GZ, ZT, LS, KX, RQ, TK, and HJ contributed to the acquisition and analysis of data. YP, LP, ZC, TK, BT, and HJ contributed to drafting the text and preparing the figures. All authors contributed to the article and approved the submitted version.

## Conflict of Interest

The authors declare that the research was conducted in the absence of any commercial or financial relationships that could be construed as a potential conflict of interest.

## Publisher’s Note

All claims expressed in this article are solely those of the authors and do not necessarily represent those of their affiliated organizations, or those of the publisher, the editors and the reviewers. Any product that may be evaluated in this article, or claim that may be made by its manufacturer, is not guaranteed or endorsed by the publisher.
