# Supplementary material for: The Natural History of Spinocerebellar Ataxia Type 3 in Mainland China: A 2-Year Cohort Study
Source: Front Aging Neurosci. 2022 Jul 5;14:917126. doi: 10.3389/fnagi.2022.917126 (PMC9294347; doi:10.3389/fnagi.2022.917126)
Supplement: Supplementary file 1 [file Data_Sheet_1.docx]

Supplementary Figures and Tables

**The natural history of spinocerebellar ataxia type 3 in mainland China: a 2-year cohort study**

Yun Peng, MD,^1#^ Linliu Peng, MD, ^1#^ Zhao Chen, MD,^1^ Huirong Peng, MD,^1^ Puzhi Wang, MD,^1^ Youming Zhang, MD,^2^ Yangping Li, PhD,^3^ Chunrong Wang, MD,^4^ Yuting Shi, MD,^1^ Xuan Hou, MD, ^1^ Zhe Long, MD,^5^ Hongyu Yuan, MD, ^1^ Na Wan, MD,^1^ Linlin Wan, MD,^1^ Keqin Xu, MD,^1^ Lijing Lei, MD,^1^ Shang Wang, MD,^1^ Lang He, MD,^1^ Yue Xie, MD,^1^ Yiqing Gong, MD,^1^ Qi Deng, MD,^1^ Guangdong Zou, MD,^1^ Zhichao Tang, MD,^1^ Lu Shen, MD, ^1, 6, 10, 11, 12^ Kun Xia, PhD,^6^ Rong Qiu, PhD,^7^ Thomas Klockgether, MD,^8, 9^ Beisha Tang, MD,^1, 6, 10, 11, 12^, Hong Jiang, MD,^1, 6, 10, 11, 12*^

#These authors have contributed equally to this work.

***Corresponding Author:**Dr. Hong Jiang, MD, Department of Neurology, Xiangya Hospital, Central South University, 87# Xiangya road, Changsha 410008,
Hunan, P.R. China. Phone: +86-731-84327216. Fax: +86-731-84327332.
Email: jianghong73868@126.com

**Numbers of Supplementary Figures:** 2

**Numbers of Supplementary Tables:** 9


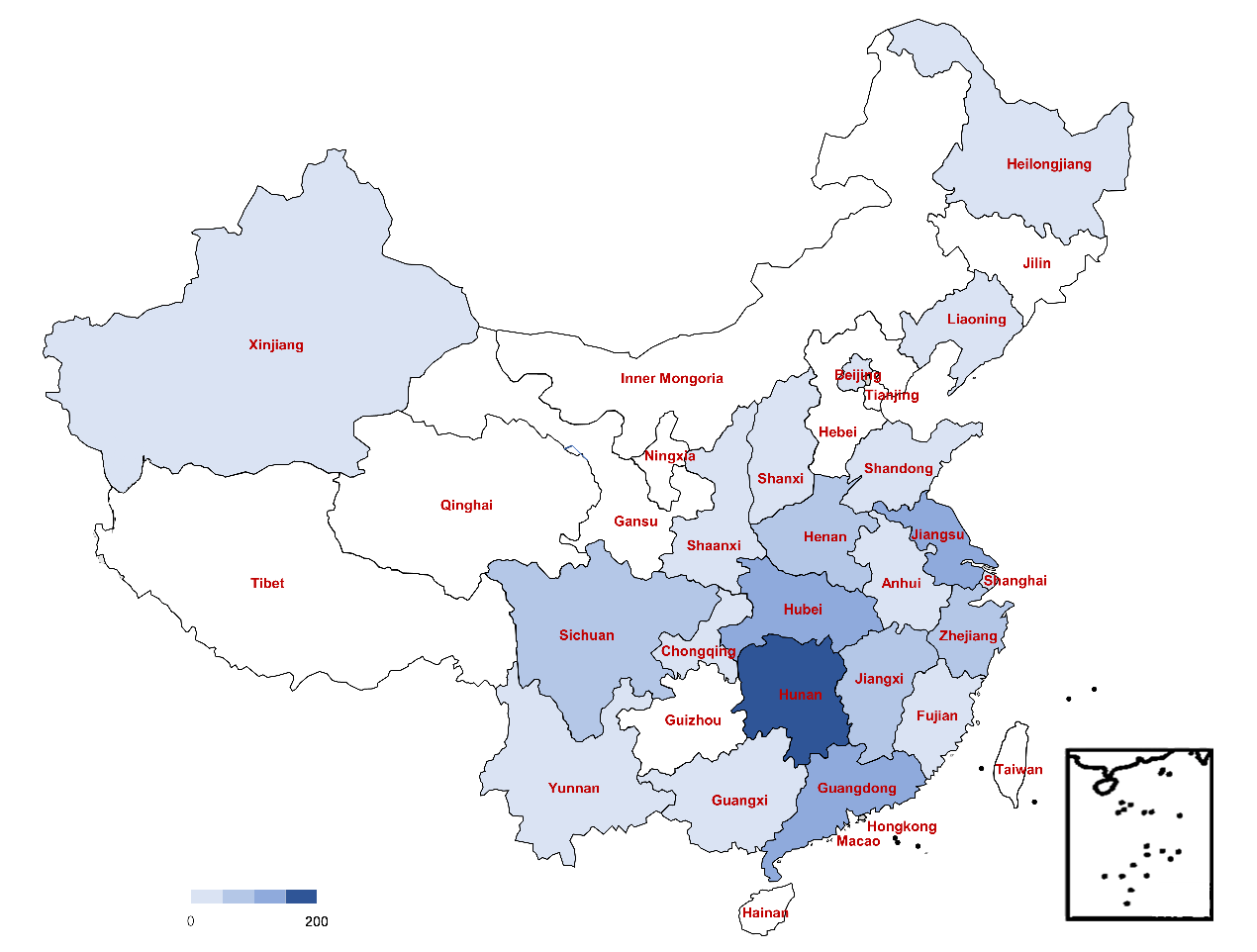


***Supplementary Figure 1 The map of the geographic locations for SCA3 patients in this study***

In this study, SCA3 patients were from 21 provinces, centrally-administered municipality, or autonomous region, and the majority (80.6%) of patients were from Hunan province in the southern China.


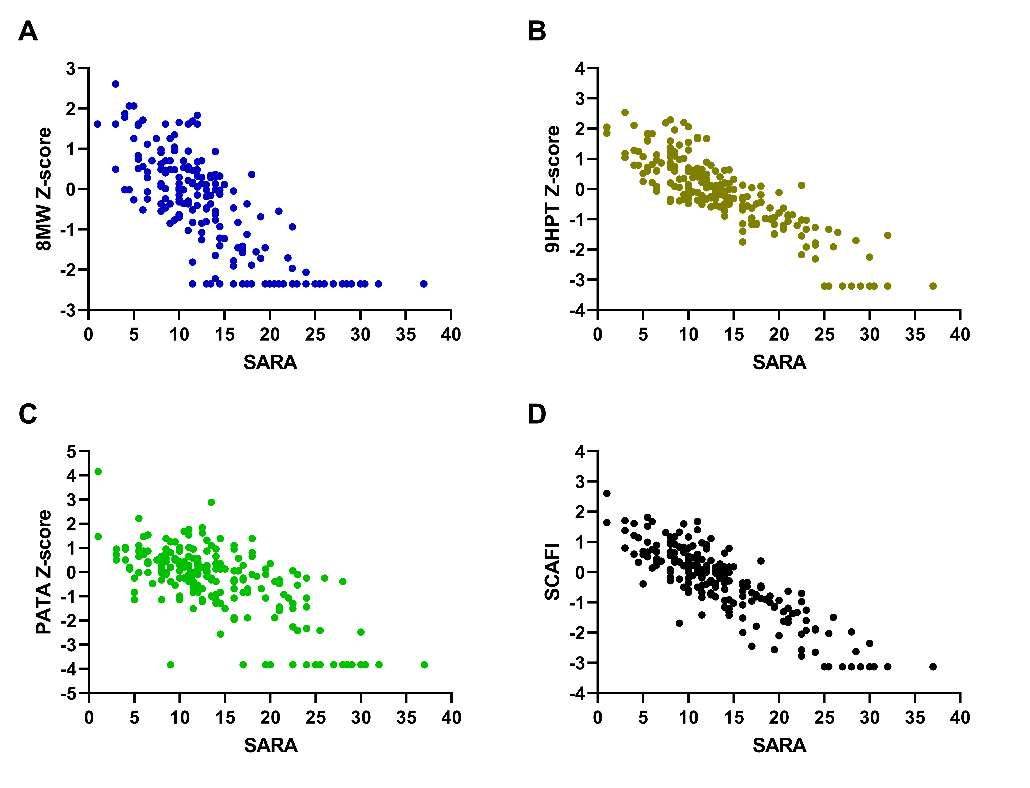


***Supplementary Figure 2 The association between SARA and SCAFI as well as its components at baseline***

The analysis was done in 204 patients with SCAFI data at baseline by using Spearman correlation analysis. The correlation coefficient was -0.79, -0.85, -0.59 and -0.83 for SARA with 8MW (figure 2A), 9HPT (figure 2B), PATA (figure 2C), and SCAFI (figure 2D), respectively. More details could be found in the *Supplementary Table 8*.

***Supplementary Table 1 Outcome measures and estimated annual progression rates***

|  | **Baseline** | | **1 year follow-up** | | | **2 years follow-up** | | | **Annual progression rate*** | | |
| --- | --- | --- | --- | --- | --- | --- | --- | --- | --- | --- | --- |
|  | Number of patients | Mean (SD) | Number of patients | Mean (SD) | SRM | Number of patients | Mean (SD) | SRM | Estimate (SE) | 95%CI | p value |
| SARA | 263 | 15.65 (7.95) | 247 | 16.93 (7.95) | 1.02 | 213 | 19.18 (8.35) | 1.14 | 1.49 (0.08) | 1.33~1.65 | <0.0001 |
| INAS | 263 | 4.84 (2.35) | 247 | 5.57 (2.32) | 0.69 | 213 | 6.13 (2.34) | 0.81 | 0.56 (0.05) | 0.47~0.66 | <0.0001 |
| SCAFI | 204 | -0.35 (1.20) | 190 | -0.63 (1.12) | -1.15 | 157 | -1.02 (1.03) | -1.57 | -0.30（0.01） | -0.33~-0.28 | <0.0001 |

*Annual progression rates of SARA and INAS were calculated on the data of 247 patients with at least one follow-up visit; and annual progression rates of SCAFI was calculated on the data of 190 patients with at least one follow-up visit and available SCAFI data. SARA = scale for the assessment and rating of ataxia; INAS= inventory of non-ataxia signs; SCAFI = SCA Functional Index; SRM = standardized response mean.

***Supplementary Table 2 Comparison of the subitem of SARA and annual progression rates between this study and two previous Taiwan studies***

|  | **This study** | | | | **The latter Taiwan study (Lin et al., 2019)** | | | | **The former Taiwan study (Lee et al., 2011)** | | | |
| --- | --- | --- | --- | --- | --- | --- | --- | --- | --- | --- | --- | --- |
|  | **Baseline** | | **Annual progression rate*** | | **Baseline** | | **Annual progression rate*** | | **Baseline** | | **Annual progression rate^#^** | |
|  | Number of patients | Mean (SD) | Estimate (SE) | 95%CI | Number of patients | Mean (SD) | Estimate (SE) | 95%CI | Number of patients | Mean (SD) | Estimate (SD) | 95%CI |
| Gait | 263 | 4.29 (2.15) | 0.28 (0.03) | 0.23~0.33 | 118 | 3.96 | 0.46 | NA | 99 | NA | 0.61 (0.55) | NA |
| Stance | 263 | 2.99 (1.72) | 0.15 (0.02) | 0.12~0.19 | 118 | 1.84 | 0.27 | NA | 99 | NA | 0.48 (0.55) | NA |
| Sitting | 263 | 0.66 (1.03) | 0.24 (0.02) | 0.19~0.29 | 118 | 0.59 | 0.14 | NA | 99 | NA | 0.27 (0.38) | NA |
| Speech disturbance | 263 | 2.05 (1.33) | 0.26 (0.02) | 0.22~0.31 | 118 | 1.51 | 0.20 | NA | 99 | NA | 0.46 (0.50) | NA |
| Finger chase | 263 | 1.17 (0.86) | 0.04 (0.01) | 0.02~0.06 | 118 | 0.95 | 0.08 | NA | 99 | NA | 0.20 (0.25) | NA |
| Nose-finger test | 263 | 0.83 (0.82) | 0.14 (0.01) | 0.11~0.17 | 118 | 0.81 | 0.12 | NA | 99 | NA | 0.24 (0.30) | NA |
| Fast alternating hand movements | 263 | 1.79 (1.10) | 0.16 (0.02) | 0.12~0.19 | 118 | 1.14 | 0.13 | NA | 99 | NA | 0.38 (0.30) | NA |
| Heel-shin slide | 263 | 1.88 (0.89) | 0.24 (0.02) | 0.20~0.28 | 118 | 1.42 | 0.15 | NA | 99 | NA | 0.36 (0.43) | NA |
| Total score | 263 | 15.65 (7.95) | 1.49 (0.08) | 1.33~1.65 | 118 | 12.22 (8.48) | 1.60 | 1.33~1.87 | 99 | 15.1 (9.2) | 3.00 (1.52) | NA |

*Annual progression rates were calculated by using linear mixed model. **^#^** Annual progression rates were calculated by dividing the score of change with the observation duration. NA = Not available. SARA = scale for the assessment and rating of ataxia.

Note: Till now, only two previous studies from a Taiwan research group have published the baseline score and progression of the subitem of SARA, named separately as the former Taiwan study(Lee et al., 2011) and the latter Taiwan study(Lin et al., 2019).

***Supplementary Table 3 Frequency of subitem of INAS in this study and EUROSCA study***

|  | This study | | | EUROSCA study paper-1  (Schmitz-Hubsch et al., 2008) | EUROSCA paper-2  (Jacobi et al., 2015) | | Comparison of Baseline between this study and EUROSCA study paper-1 |
| --- | --- | --- | --- | --- | --- | --- | --- |
|  | Baseline  (n=263) | 1-year  (n=247) | 2-year  (n=213) | Baseline  (n=139) | Baseline  (n=139) | 3-year  (n=92) | p-value |
| Hyperreflexia | 50.6 | 52.6 | 48.4 | 40.1 | 36.9 | 38.1 | 0.049 |
| Areflexia | 58.6 | 63.2 | 69.0 | 57.8 | 59.3 | 64.0 | 0.847 |
| Extensor plantar | 56.7 | 64.8 | 69.0 | 41.9 | 38.8 | 37.6 | 0.004 |
| Spasticity | 54.0 | 70.9 | 72.8 | 44.4 | 43.9 | 52.4 | 0.073 |
| Paresis | 20.2 | 24.7 | 33.8 | 24.8 | 21.2 | 45.9 | 0.318 |
| Muscle atrophy | 46.8 | 51.4 | 60.6 | 39.0 | 35.3 | 47.1 | 0.128 |
| Fasciculations | 50.2 | 54.7 | 54.5 | 37.0 | 33.7 | 48.8 | 0.010 |
| Myoclonus | 22.8 | 26.7 | 26.8 | 4.4 | 3.5 | 8.1 | **2.00e-6** |
| Rigidity | 7.2 | 6.1 | 5.6 | 10.3 | 8.3 | 10.7 | 0.323 |
| Chorea/dyskinesia | 0.0 | 0.0 | 0.5 | 10.1 | 4.7 | 8.1 | **1.62e-7** |
| Dystonia | 16.3 | 15.8 | 16.4 | 23.9 | 18.6 | 22.1 | 0.072 |
| Resting tremor | 2.3 | 1.2 | 1.9 | 3.6 | 1.2 | 1.2 | 0.442 |
| Sensory symptoms | 20.2 | 24.7 | 27.2 | 65.6 | 70.1 | 85.7 | **2.01e-19** |
| Urinary dysfunction | 34.6 | 38.1 | 51.2 | 45.6 | 51.2 | 64.3 | 0.035 |
| Cognitive impairment | 11.0 | 12.1 | 13.6 | 19.3 | 13.4 | 18.3 | 0.021 |
| Brainstem oculomotor signs | 32.3 | 49.4 | 62.4 | 67.9 | 50.6 | 69.9 | **1.25e-11** |

*Data are given as percentages (%). The frequency of subitem of INAS at baseline were compared between this study and EUROSCA study paper-1 by chi-square (χ^2^) test. After Bonferroni correction, only p values < 0.0031 were considered significant. INAS = inventory of non-ataxia signs.

Note: Till now, only the EUROSCA study published two papers about the subitem of INAS, which we named as the EUROSCA paper-1 (Schmitz-Hubsch et al., 2008) and EUROSCA paper-2 (Jacobi et al., 2015). The EUROSCA paper-1 shows the frequency of subitem of INAS at baseline in the main manuscript, and the EUROSCA paper-2 shows the frequency of subitem of INAS at baseline and 3-year timepoint in its supplementary file. However, the frequency of subitem of INAS at baseline shown in these two papers are contradictory. After checking with statistician, we thought the data of EUROSCA paper-1 should be correct. Therefore, we compare the frequency of subitem of INAS at baseline between this study and EUROSCA study paper-1.

***Supplementary Table 4 Linear mixed effect modeling for potential factors affecting SARA progression***

| **Step1: Univariate analysis (Model 1~7)** | | | | | | |
| --- | --- | --- | --- | --- | --- | --- |
| **SARA-Model 1** | | | | | | |
| **AIC** | **Fixed effect** | **Estimate** | **SE** | **95% CI** | **t-value** | **p-value** |
| 3117.746 | Intercept | 0.002 | 1.041 | -2.930~2.935 | 0.002 | 1.000 |
|  | **Time** | **1.491** | **0.787** | **1.336~1.646** | **18.944** | **4.266e-50** |
|  | **Time* Length of expanded allele** | **0.107** | **0.024** | **0.060~0.154** | **4.463** | **1.2e-5** |
| **SARA-Model 2** | | | | | | |
| **AIC** | **Fixed effect** | **Estimate** | **SE** | **95% CI** | **t-value** | **p-value** |
| 3134.006 | Intercept | 0.005 | 0.500 | -0.983~0.993 | 0.010 | 0.992 |
|  | **Time** | **1.485** | **0.082** | **1.324~1.646** | **18.156** | **1.975e-47** |
|  | Time* Length of normal allele | 0.003 | 0.012 | -0.022~0.027 | 0.216 | 0.829 |
| **SARA-Model 3** | | | | | | |
| **AIC** | **Fixed effect** | **Estimate** | **SE** | **95% CI** | **t-value** | **p-value** |
| 3131.866 | Intercept | 0.002 | 0.499 | -0.985~0.989 | 0.003 | 0.997 |
|  | **Time** | **1.578** | **0.119** | **1.343~1.812** | **13.244** | **1.238e-30** |
|  | Time*Gender | -0.167 | 0.164 | -0.489~0.155 | -1.021 | 0.308 |
| **SARA-Model 4** | | | | | | |
| **AIC** | **Fixed effect** | **Estimate** | **SE** | **95% CI** | **t-value** | **p-value** |
| 3246.776 | Intercept | 0.205 | 0.490 | -0.759~1.170 | 0.419 | 0.675 |
|  | **Time** | **1.494** | **0.080** | **1.336~1.652** | **18.655** | **4.396e-49** |
|  | **Time*Age at baseline** | **-0.027** | **0.007** | **-0.042~-0.012** | **-3.634** | **3.39e-4** |
| **SARA-Model 5** | | | | | | |
| **AIC** | **Fixed effect** | **Estimate** | **SE** | **95% CI** | **t-value** | **p-value** |
| 3244.681 | Intercept | 0.205 | 3.724 | -9.947~10.358 | 0.055 | 1.000 |
|  | **Time** | **1.492** | **0.080** | **1.335~1.648** | **18.752** | **2.003e-49** |
|  | **Time*Age at onset** | **-0.030** | **0.008** | **-0.046~-0.014** | **-3.735** | **2.33e-4** |
| **SARA-Model 6** | | | | | | |
| **AIC** | **Fixed effect** | **Estimate** | **SE** | **95% CI** | **t-value** | **p-value** |
| 3256.820 | Intercept | 0.204 | 0.490 | -0.761~1.169 | 0.416 | 0.678 |
|  | **Time** | **1.488** | **0.082** | **1.327~1.650** | **18.199** | **1.426e-47** |
|  | Time*Disease duration at baseline | 0.006 | 0.016 | -0.026~0.038 | 0.369 | 0.712 |
| **SARA-Model 7** | | | | | | |
| **AIC** | **Fixed effect** | **Estimate** | **SE** | **95% CI** | **t-value** | **p-value** |
| 1839.967 | Intercept | 0.015 | 0.032 | -0.049~0.079 | 0.460 | 0.646 |
|  | **Time** | **1.489** | **0.082** | **1.327~1.650** | **18.199** | **1.422e-47** |
|  | **SARA at baseline** | **0.998** | **0.004** | **0.990~1.006** | **245.502** | **0.000** |
|  | Time*SARA at baseline | 0.001 | 0.010 | -0.012~0.021 | 0.061 | 0.951 |
| **Step2: Multivariate analysis (Model 8~9)** | | | | | | |
| **SARA-Model 8 (multivariable model)** | | | | | | |
| **AIC** | **Fixed effect** | **Estimate** | **SE** | **95% CI** | **t-value** | **p-value** |
| 1851.459 | Intercept | 0.015 | 50.898 | -99.742~99.773 | 0.000 | 1.000 |
|  | **Time** | **1.489** | **0.079** | **1.333~1.646** | **18.781** | **1.647e-48** |
|  | **SARA at baseline** | **0.998** | **0.005** | **0.989~1.008** | **203.682** | **4.256e-126** |
|  | **Time*Length of expanded allele** | **0.088** | **0.036** | **0.017~0.159** | **2.451** | **0.015** |
|  | Time*Age at baseline | 0.009 | 0.016 | -0.022~0.041 | 0.598 | 0.551 |
|  | Time*Age at onset | -0.017 | 0.018 | -0.053~0.018 | -0.948 | 0.344 |
| **SARA-Model 9 (multivariable model) (Final model)** | | | | | | |
| **AIC** | **Fixed effect** | **Estimate** | **SE** | **95% CI** | **t-value** | **p-value** |
| 1818.997 | Intercept | 0.015 | 0.032 | -0.049~0.079 | 0.464 | 0.643 |
|  | **Time** | **1.491** | **0.079** | **1.336~1.646** | **18.938** | **4.398e-50** |
|  | **SARA at baseline** | **0.999** | **0.004** | **0.991~1.007** | **245.779** | **0.000** |
|  | **Time*Length of expanded allele** | **0.107** | **0.024** | **0.060~0.154** | **4.493** | **1.1e-5** |

SARA = scale for the Assessment and Rating of Ataxia; SE = standard error; 95% CI = 95% confidence interval; AIC = Akaike information criterion. Length of expanded allele or normal allele refers to the CAG repeats in the *ATXN3* gene.

For exploring the possible factors affecting the progression of SARA, we tested 7 candidate variables. Specifically, at the first step (Univariate analysis, model 1~7), we used the SARA score as dependent variables, and used repeat length of expanded allele, repeat length of normal allele, gender, age at baseline, age at onset, and disease duration at baseline as independent variables. Influence of these variables on the progression rate of SARA were tested via interactions between corresponding variables and time variable. In addition, we included the baseline SARA scores as main effects. Then, at the second step (Multivariate analysis, model 8~9), independent factors that were significant in the univariate analysis were included in a multivariate model, and the final model were determined with backward selection. The significance of candidate variable and the AIC value of the model were used for optimizing models.

The time variable was the year since inclusion. Numerical variables were mean centred to help interpretation, and family was included as a random factor in all these models.

***Supplementary Table 5 Linear mixed effect modeling for potential factors affecting INAS progression***

| Step1: Univariate analysis (Model 1~7) | | | | | | |
| --- | --- | --- | --- | --- | --- | --- |
| **INAS-Model 1** | | | | | | |
| **AIC** | **Fixed effect** | **Estimate** | **SE** | **95% CI** | **t-value** | **p-value** |
| 2349.697 | Intercept | 0.015 | 0.337 | -2.557~2.587 | 0.044 | 1.000 |
|  | **Time** | **0.564** | **0.048** | **0.469~0.659** | **11.662** | **3.104e-25** |
|  | **Time* Length of expanded allele** | **0.027** | **0.014** | **0.0001~0.055** | **1.977** | **0.049** |
| **INAS-Model 2** | | | | | | |
| **AIC** | **Fixed effect** | **Estimate** | **SE** | **95% CI** | **t-value** | **p-value** |
| 2334.536 | Intercept | 0.038 | 0.239 | -1.727~1.803 | 0.160 | 1.000 |
|  | **Time** | **0.561** | **0.048** | **0.467~0.655** | **11.721** | **1.712e-25** |
|  | Time* Length of normal allele | 0.0004 | 0.006 | -0.013~0.014 | 0.066 | 0.948 |
| **INAS-Model 3** | | | | | | |
| **AIC** | **Fixed effect** | **Estimate** | **SE** | **95% CI** | **t-value** | **p-value** |
| 2349.206 | Intercept | 0.017 | 0.168 | -0.769~0.804 | 0.102 | 1.000 |
|  | **Time** | **0.593** | **0.068** | **0.459~0.727** | **8.721** | **3.402e-16** |
|  | Time*Gender | -0.056 | 0.091 | -0.234~0.123 | -0.614 | 0.540 |
| **INAS-Model 4** | | | | | | |
| **AIC** | **Fixed effect** | **Estimate** | **SE** | **95% CI** | **t-value** | **p-value** |
| 2354.762 | Intercept | 0.017 | 0.181 | -0.545~0.578 | 0.092 | 1.000 |
|  | **Time** | **0.565** | **0.048** | **0.470~0.660** | **11.753** | **1.320e-25** |
|  | Time*Age at baseline | -0.004 | 0.004 | -0.012~0.004 | -0.974 | 0.331 |
| **INAS-Model 5** | | | | | | |
| **AIC** | **Fixed effect** | **Estimate** | **SE** | **95% CI** | **t-value** | **p-value** |
| 2351.21 | Intercept | 0.015 | 0.158 | -0.297~0.326 | 0.092 | 0.927 |
|  | **Time** | **0.565** | **0.048** | **0.470~0.660** | **11.760** | **1.316e-25** |
|  | **Time*Age at onset** | **-0.010** | **0.005** | **-0.019~-0.001** | **-2.112** | **0.036** |
| **INAS-Model 6** | | | | | | |
| **AIC** | **Fixed effect** | **Estimate** | **SE** | **95% CI** | **t-value** | **p-value** |
| 2350.118 | Intercept | 0.017 | 0.158 | -0.532~0.566 | 0.108 | 1.000 |
|  | **Time** | **0.557** | **0.049** | **0.461~0.653** | **11.455** | **1.704e-24** |
|  | **Time*Disease duration at baseline** | **0.019** | **0.009** | **0.002~0.036** | **2.177** | **0.030** |
| **INAS-Model 7** | | | | | | |
| **AIC** | **Fixed effect** | **Estimate** | **SE** | **95% CI** | **t-value** | **p-value** |
| 1843.468 | **Intercept** | **0.152** | **0.059** | **0.035~0.269** | **2.572** | **0.011** |
|  | **Time** | **0.547** | **0.046** | **0.457~0.638** | **11.898** | **4.951e-26** |
|  | **INAS at baseline** | **0.953** | **0.023** | **0.909~0.998** | **42.149** | **1.298e-122** |
|  | **Time*INAS at baseline** | **-0.099** | **0.020** | **-0.138~-0.060** | **-4.999** | **1e-6** |
| Step2: Multivariate analysis (Model 8~10) | | | | | | |
| **INAS-Model 8 (multivariable model)** | | | | | | |
| **AIC** | **Fixed effect** | **Estimate** | **SE** | **95% CI** | **t-value** | **p-value** |
| 1856.918 | Intercept | 0.152 | 0.059 | 0.035~0.268 | 2.572 | 0.011 |
|  | **Time** | **0.538** | **0.046** | **0.448~0.628** | **11.768** | **1.404e-25** |
|  | **INAS at baseline** | **0.953** | **0.023** | **0.908~0.998** | **42.113** | **1.768e-112** |
|  | **Time*INAS at baseline** | **-0.124** | **0.022** | **-0.166~-0.081** | **-5.706** | **3.341e-8** |
|  | Time* Length of expanded allele | 0.025 | 0.022 | -0.018~0.069 | 1.139 | 0.256 |
|  | Time*Age at onset | -0.003 | 0.007 | -0.017~0.011 | -0.454 | 0.650 |
|  | **Time*Disease duration at baseline** | 0.020 | 0.010 | 0.001~0.039 | 2.121 | 0.035 |
| **INAS-Model 9 (multivariable model)** | | | | | | |
| **AIC** | **Fixed effect** | **Estimate** | **SE** | **95% CI** | **t-value** | **p-value** |
| 1847.409 | Intercept | 0.152 | 2255.99 | -4421.5~4421.8 | 0.000 | 1.000 |
|  | **Time** | 0.539 | 0.046 | 0.449~0.630 | 11.741 | 1.5929e-25 |
|  | **INAS at baseline** | 0.953 | 0.023 | 0.908~0.998 | 42.123 | 1.635e-122 |
|  | **Time*INAS at baseline** | -0.111 | 0.021 | -0.152~-0.070 | -5.364 | 1.9011e-7 |
|  | Time*Disease duration at baseline | 0.018 | 0.009 | -0.0005~0.036 | 1.918 | 0.056 |
| **INAS-Model 10 (multivariable model) (Final model)** | | | | | | |
| **AIC** | **Fixed effect** | **Estimate** | **SE** | **95% CI** | **t-value** | **p-value** |
| 1843.468 | **Intercept** | **0.152** | **0.059** | **0.035~0.269** | **2.572** | **0.011** |
|  | **Time** | **0.547** | **0.046** | **0.457~0.638** | **11.898** | **4.9509e-26** |
|  | **INAS at baseline** | **0.953** | **0.023** | **0.909~0.998** | **42.149** | **1.298e-122** |
|  | **Time*INAS at baseline** | **-0.099** | **0.020** | **-0.138~-0.060** | **-4.999** | **1e-6** |

INAS= inventory of non-ataxia signs; SE = standard error; 95% CI = 95% confidence interval; AIC = Akaike information criterion. Length of expanded allele or normal allele refers to the CAG repeats in the *ATXN3* gene.

For exploring the possible factors affecting the progression of INAS, we tested 7 candidate variables. Specifically, at the first step (Univariate analysis, model 1~7), we used the INAS score as dependent variables, and used repeat length of expanded allele, repeat length of normal allele, gender, age at baseline, age at onset, and disease duration at baseline as independent variables. Influence of these variables on the progression rate of INAS were tested via interactions between corresponding variables and time variable. In addition, we included the baseline INAS scores as main effects. Then, at the second step (Multivariate analysis, model 8~10), independent factors that were significant in the univariate analysis were included in a multivariate model, and the final model were determined with backward selection. The significance of candidate variable and the AIC value of the model were used for optimizing models.

The time variable was the year since inclusion. Numerical variables were mean centred to help interpretation, and family was included as a random factor in all these models.

***Supplementary Table 6 Linear mixed effect modeling for potential factors affecting SCAFI progression***

| **Step1: Univariate analysis (Model 1~7)** | | | | | | |
| --- | --- | --- | --- | --- | --- | --- |
| **SCAFI-Model 1** | | | | | | |
| **AIC** | **Fixed effect** | **Estimate** | **SE** | **95% CI** | **t-value** | **p-value** |
| 556.477 | Intercept | 0.314 | 2927.771 | -5738.0~5738.6 | 0.0001 | 1.000 |
|  | **Time** | **-0.303** | **0.013** | **-0.329~-0.277** | **-22.806** | **8.487e-55** |
|  | Time* Length of expanded allele | -0.0009 | 0.003 | -0.007~0.005 | -0.311 | 0.756 |
| **SCAFI-Model 2** | | | | | | |
| **AIC** | **Fixed effect** | **Estimate** | **SE** | **95% CI** | **t-value** | **p-value** |
| 553.686 | Intercept | 0.315 | 0.086 | 0.146~0.483 | 3.681 | 0.0003 |
|  | **Time** | **-0.303** | **0.013** | **-0.329~-0.277** | **-23.097** | **2.259e-56** |
|  | **Time* Length of normal allele** | **0.004** | **0.002** | **0.0002~0.007** | **2.111** | **0.036** |
| **SCAFI-Model 3** | | | | | | |
| **AIC** | **Fixed effect** | **Estimate** | **SE** | **95% CI** | **t-value** | **p-value** |
| 552.513 | Intercept | 0.314 | 0.087 | 0.142~0.485 | 3.615 | 0.0004 |
|  | **Time** | **-0.308** | **0.017** | **-0.342~-0.274** | **-18.018** | **9.233e--45** |
|  | Time*Gender | 0.010 | 0.210 | -0.032~0.051 | 0.463 | 0.644 |
| **SCAFI-Model 4** | | | | | | |
| **AIC** | **Fixed effect** | **Estimate** | **SE** | **95% CI** | **t-value** | **p-value** |
| 558.730 | Intercept | 0.314 | 0.100 | -0.138~0.766 | 3.132 | 1.000 |
|  | **Time** | **-0.303** | **0.013** | **-0.329~-0.277** | **-22.817** | **2.155e-55** |
|  | Time*Age at baseline | 4.100e-6 | 0.001 | -0.002~0.002 | 0.004 | 0.997 |
| **SCAFI-Model 5** | | | | | | |
| **AIC** | **Fixed effect** | **Estimate** | **SE** | **95% CI** | **t-value** | **p-value** |
| 558.449 | Intercept | 0.314 | 0.087 | 0.142~0.486 | 3.614 | 0.0004 |
|  | **Time** | **-0.303** | **0.013** | **-0.329~-0.277** | **-22.818** | **2.577e-55** |
|  | Time*Age at onset | 0.0004 | 0.001 | -0.002~0.003 | 0.439 | 0.661 |
| **SCAFI-Model 6** | | | | | | |
| **AIC** | **Fixed effect** | **Estimate** | **SE** | **95% CI** | **t-value** | **p-value** |
| 556.189 | Intercept | 0.314 | 1793.486 | -3514.8~3515.5 | 0.0002 | 1.000 |
|  | **Time** | **-0.305** | **0.014** | **-0.332~-0.278** | **-22.476** | **3.480e-51** |
|  | Time*Disease duration at baseline | -0.003 | 0.002 | -0.007~0.002 | -1.198 | 0.233 |
| **SCAFI-Model 7** | | | | | | |
| **AIC** | **Fixed effect** | **Estimate** | **SE** | **95% CI** | **t-value** | **p-value** |
| -332.462 | Intercept | -0.007 | 20.378 | -39.948~39.934 | 0.0003 | 1.000 |
|  | **Time** | **-0.272** | **0.011** | **-0.293~-0.250** | **-25.198** | **8.449e-60** |
|  | **SCAFI at baseline** | **0.996** | **0.007** | **0.982~1.010** | **139.931** | **3.341e-195** |
|  | **Time*SCAFI at baseline** | **-0.094** | **0.009** | **-0.112~-0.077** | **-10.653** | **9.676e-21** |
| **Step2: Multivariate analysis (Model 8)** | | | | | | |
| **SCAFI-Model 8 (multivariable model) (Final model)** | | | | | | |
| **AIC** | **Fixed effect** | **Estimate** | **SE** | **95% CI** | **t-value** | **p-value** |
| -325.548 | Intercept | -0.007 | 0.010 | -0.027~0.013 | -0.671 | 0.502 |
|  | **Time** | **-0.272** | **0.011** | **-0.293~-0.251** | **-25.391** | **4.061e-60** |
|  | **SCAFI at baseline** | **0.996** | **0.007** | **0.982~1.010** | **139.931** | **3.340e-195** |
|  | **Time* Length of normal allele** | **0.004** | **0.002** | **0.0002~0.0071** | **2.060** | **0.041** |
|  | **Time*SCAFI at baseline** | **-0.094** | **0.009** | **-0.111~-0.076** | **-10.669** | **9.163e-21** |

SCAFI = SCA Functional Index; SE = standard error; 95% CI = 95% confidence interval; AIC = Akaike information criterion. Length of expanded allele or normal allele refers to the CAG repeats in the *ATXN3* gene.

For exploring the possible factors affecting the progression of SCAFI, we tested 7 candidate variables. Specifically, at the first step (Univariate analysis, model 1~7), we used the SCAFI score as dependent variables, and used repeat length of expanded allele, repeat length of normal allele, gender, age at baseline, age at onset, and disease duration at baseline as independent variables. Influence of these variables on the progression rate of SCAFI were tested via interactions between corresponding variables and time variable. In addition, we included the baseline SCAFI scores as main effects. Then, at the second step (Multivariate analysis, model 8), independent factors that were significant in the univariate analysis were included in a multivariate model, and the final model were determined with backward selection. The significance of candidate variable and the AIC value of the model were used for optimizing models.

The time variable was the year since inclusion. Numerical variables were mean centred to help interpretation, and family was included as a random factor in all these models.

***Supplementary Table 7 Demographic and clinical characteristics of male and female participants at baseline***

|  | **Male** | **Female** | **Statistics (*U*)** | ***P*** |
| --- | --- | --- | --- | --- |
| Sample size | 125 | 138 | NA | NA |
| Age at baseline (years) | 44.26 ± 10.77 | 45.05 ± 11.76 | 8325.50 | 0.627 |
| Age at onset (years) | 35.86 ± 9.77 | 36.68 ± 10.63 | 8110.00 | 0.403 |
| Disease duration (years) | 8.39 ± 4.63 | 8.37 ± 5.47 | 8319.50 | 0.619 |
| Length of expanded allele (repeat units) | 71.81 ± 3.45 | 71.95 ± 3.42 | 8510.50 | 0.852 |
| Length of normal allele (repeat units) ^a^ | 20.52 ± 6.09 | 19.49 ± 5.81 | 7586.00 | 0.104 |
| SARA score | 15.03 ± 7.42 | 16.21 ± 8.38 | 7988.00 | 0.301 |
| INAS count | 4.55 ± 2.16 | 5.10 ± 2.48 | 7594.00 | 0.091 |
| SCAFI score ^b^ | -0.14 ± 1.11 | -0.54 ± 1.25 | 4286.00 | 0.032 |

A total of 263 SCA3 patients were divided into Male and Female subgroup. Quantitative variables are given as mean ± SD. ^a^ There was a patient with homozygous *ATXN3* mutation (with 67 CAG repeats on both allele), and this patient was excluded when calculating the mean and SD for length of normal allele (repeat units). ^b^ SCAFI score was available in 204 patients (Male:97; Female:107) of the full cohort.

The Mann-Whitney *U* test was used for the comparation of quantitative variables between groups, including age at baseline, age at onset, disease duration, length of expanded allele, length of normal allele, SARA score, INAS count, and SCAFI. After Bonferroni correction, only *P* values < 0.0062 were considered significant.

***Supplementary Table 8 Association between SARA and SCAFI as well as its components at baseline***

|  | 8MW Z-score | | 9HPT Z-score | | PATA Z-score | | SCAFI | |
| --- | --- | --- | --- | --- | --- | --- | --- | --- |
|  | r_s_ | *P* | r_s_ | *P* | r_s_ | *P* | r_s_ | *P* |
| SARA | -0.79 | < 0.001 | -0.85 | < 0.001 | -0.59 | < 0.001 | -0.83 | < 0.001 |

The analysis was done in 204 patients with SCAFI data at baseline by using Spearman correlation analysis. After Bonferroni correction, only *P* values < 0.0125 were considered significant.

***Supplementary Table 9 Baseline demographic and clinical characteristics of participants with missing data of SCAFI and participants fully investigated***

|  | **Participants with missing data** | **Participants fully investigated** | **Statistics (χ^2^ or *U*)** | ***P*** |
| --- | --- | --- | --- | --- |
| Sample size | 106 | 157 | NA | NA |
| Women (%) | 57 (53.8%) | 81 (51.6%) | 0.121 | 0.728 |
| Age at baseline (years) | 45.63 ± 12.85 | 44.03 ± 10.08 | 7657.5 | 0.273 |
| Age at onset (years) | 36.43 ± 11.02 | 36.20 ± 9.68 | 8314.5 | 0.991 |
| Disease duration (years) | 9.20 ± 5.39 | 7.83 ± 4.79 | 7022.0 | 0.031 |
| Length of expanded allele (repeat units) | 72.01 ± 3.46 | 71.80 ± 3.42 | 8119.5 | 0.738 |
| Length of normal allele (repeat units) ^a^ | 19.95 ± 6.05 | 19.99 ± 5.90 | 8122.5 | 0.838 |
| SARA score | 17.79 ± 8.80 | 14.21 ± 6.98 | 6181.5 | **0.0004** |
| INAS count | 5.08 ± 2.19 | 4.68 ± 2.44 | 7260.5 | 0.077 |

A total of 263 SCA3 patients were divided into two subgroups: participants with missing data, and participants fully investigated. Quantitative variables are given as mean ± SD; categorical variables as given as numbers (frequency); SARA = scale for the Assessment and Rating of Ataxia; INAS= inventory of non-ataxia signs. Length of expanded allele or normal allele refers to the CAG repeats in the *ATXN3* gene. ^a^ There was a patient with homozygous *ATXN3* mutation (with 67 CAG repeats on both allele), and this patient was excluded when calculating the mean and SD for length of normal allele (repeat units). The Mann-Whitney *U* test was used for the comparation of quantitative variables between groups, including age at baseline, age at onset, disease duration, length of expanded allele, length of normal allele, SARA score, and INAS count. The χ^2^ test for the comparation of gender between groups. After Bonferroni correction, only *P* values < 0.00625 were considered significant.

**Reference**

Jacobi, H., Du Montcel, S.T., Bauer, P., Giunti, P., Cook, A., Labrum, R., et al. (2015). Long-term disease progression in spinocerebellar ataxia types 1, 2, 3, and 6: a longitudinal cohort study. *Lancet Neurol* 14**,** 1101-1108.

Lee, Y.C., Liao, Y.C., Wang, P.S., Lee, I.H., Lin, K.P., and Soong, B.W. (2011). Comparison of cerebellar ataxias: A three-year prospective longitudinal assessment. *Mov Disord* 26**,** 2081-2087.

Lin, Y.C., Lee, Y.C., Hsu, T.Y., Liao, Y.C., and Soong, B.W. (2019). Comparable progression of spinocerebellar ataxias between Caucasians and Chinese. *Parkinsonism Relat Disord* 62**,** 156-162.

Schmitz-Hubsch, T., Coudert, M., Bauer, P., Giunti, P., Globas, C., Baliko, L., et al. (2008). Spinocerebellar ataxia types 1, 2, 3, and 6: disease severity and nonataxia symptoms. *Neurology* 71**,** 982-989.
